# Data on antibiogram and resistance genes of *Enterobacterales* isolated from fresh vegetables in Ecuador

**DOI:** 10.1016/j.dib.2022.108249

**Published:** 2022-05-11

**Authors:** Gabriela Barragán-Fonseca, Jessica Tubón, William Calero-Cáceres

**Affiliations:** UTA-RAM-One Health, Department of Food and Biotechnology Science and Engineering, Universidad Técnica de Ambato, Ambato, Ecuador

**Keywords:** Antibiotic resistance, Raw vegetables, Antibiogram, *Enterobacteriaceae*, PCR analysis

## Abstract

This article describes the occurrence, antibiograms, and detection of antibiotic resistance genes of *Enterobacterales* isolated from fresh vegetables commercialized in Riobamba, Ecuador. *Escherichia coli* isolates were screened to detect diarrheagenic pathotypes via PCR. Agar diffusion assay was performed to determine the phenotypic antibiotic resistance of the *Enterobacterales* strains. The presence of antibiotic resistance genes conferring resistance against beta-lactams, mobile colistin resistance, carbapenems, quinolones, tetracyclines, and sulphonamides was detected via PCR amplification.

## Specifications Table

**Table:** 

Subject	Microbiology: Bacteriology
Specific subject area	Food microbiology, antimicrobial resistance.
Type of data	Table and figure
How the data were acquired	Bacteria culture (Chromocult Agar and MacConkey Agar, Merck KGaA, Darmstadt, Germany, ChromAgar mSuperCARBA. Chromagar, France), confirmation of bacteria by biochemical tests: a catalase test using 30% hydrogen peroxide (Merck Millipore, Darmstadt, Germany); triple sugar iron agar test (TSI) (Becton Dickinson GmbH, Heidelberg, Germany), Simmons citrate agar test (Merck, Darmstadt, Germany), Christensen urea agar test (Britania Lab., Buenos Aires, Argentina), indole reaction using tryptone water (Merck, Darmstadt, Germany) and Kovac's reagent (Sigma Aldrich, St. Louis, USA). PCR detection (using a thermocycler Labnet MultiGeneTM Gradient, Labnet International, USA). DNA extraction by thermal shock. Agar gel electrophoresis (using a transilluminator Enduro GDS Touch, Labnet International, USA).
Data format	Analyzed
Description of data collection	Enterobacterales species were obtained from fresh vegetables in markets of Riobamba, Ecuador. The bacteria were isolated using both Chromocult®, Mac Conkey, and ChromAgar mSuperCARBA Agar without antibiotic pressure. Biochemical tests were analyzed using ABIS Automated Biometric Identification System software. Antimicrobial susceptibility testing was performed using the disc-diffusion method on Mueller-Hinton agar plates according to the National Committee for Clinical Laboratory Standards. All data was captured into Microsoft Excel®. Pie charts were created using Microsoft Excel®. Heatmaps of antibiotic resistance profiles and hierarchical clustering were performed using Multiexperiment Viewer software version 4.8.1.
Data source location	Technical University of Ambato, Ambato, Ecuador.
Data accessibility	All data are presented in this article.

## Value of the Data

• This data contributes information about the antibiotic resistance profiles on *Enterobacterales strains* isolated from raw vegetables that will facilitate pathogen surveillance in Ecuador and Latin America.
• This data is useful for the scientific community to determine the presence of pathogenic *E. coli* isolates and antibiotic resistance genes, including mobile colistin resistance genes, carbapenemases, quinolone resistance genes, and extended-spectrum beta-lactamases present on *Enterobacterales* strains isolated from raw vegetables.
• Researchers and policymakers involved with the work related to the One Health initiative could also benefit from this data for retrospective and comparative analysis or epidemiological surveillance projects.

## 1 Data Description

**Fig. 1:**
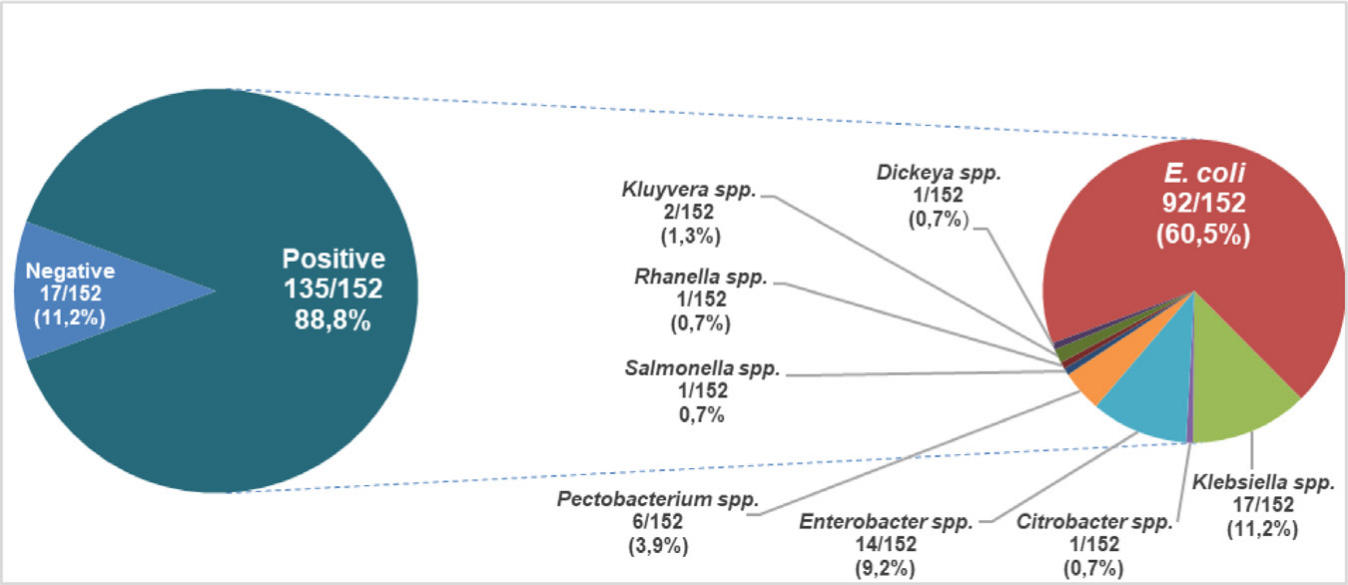
Occurrence of *Enterobacterales* on 152 samples of fresh vegetables in Riobamba, Ecuador.

The data presented show the frequency of isolation of *Enterobacterales* in 152 samples of fresh vegetables in Riobamba, Ecuador (Fig 1). The specific characteristics (date of sampling, type of vegetable, location) of the samples were reported in supplementary Table S1. One or two isolates from each sample were analyzed to avoid the isolation of clonal bacteria. One hundred ninety-three isolates were analyzed, the results of the isolates, samples and their biochemical tests were reported in supplementary Table S2. Among them, 97 isolates correspond to *Escherichia coli* and 96 isolates to other *Enterobacterales*.

**Fig 2:**
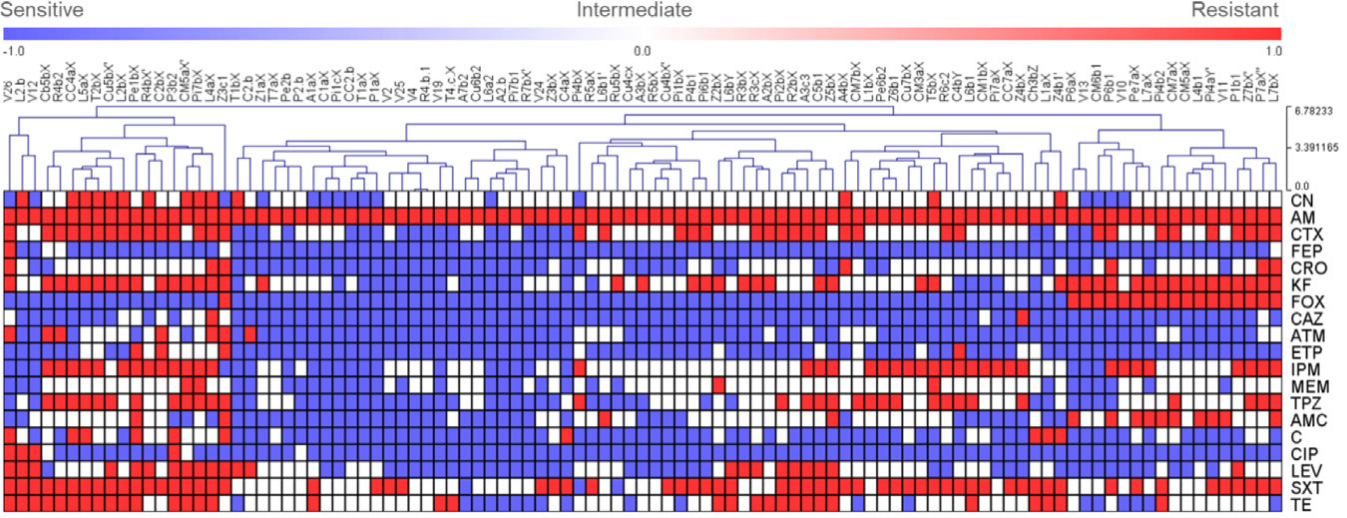
Profiles of antibiotic resistance and a hierarchical tree of *E. coli* isolates.

**Fig 3:**
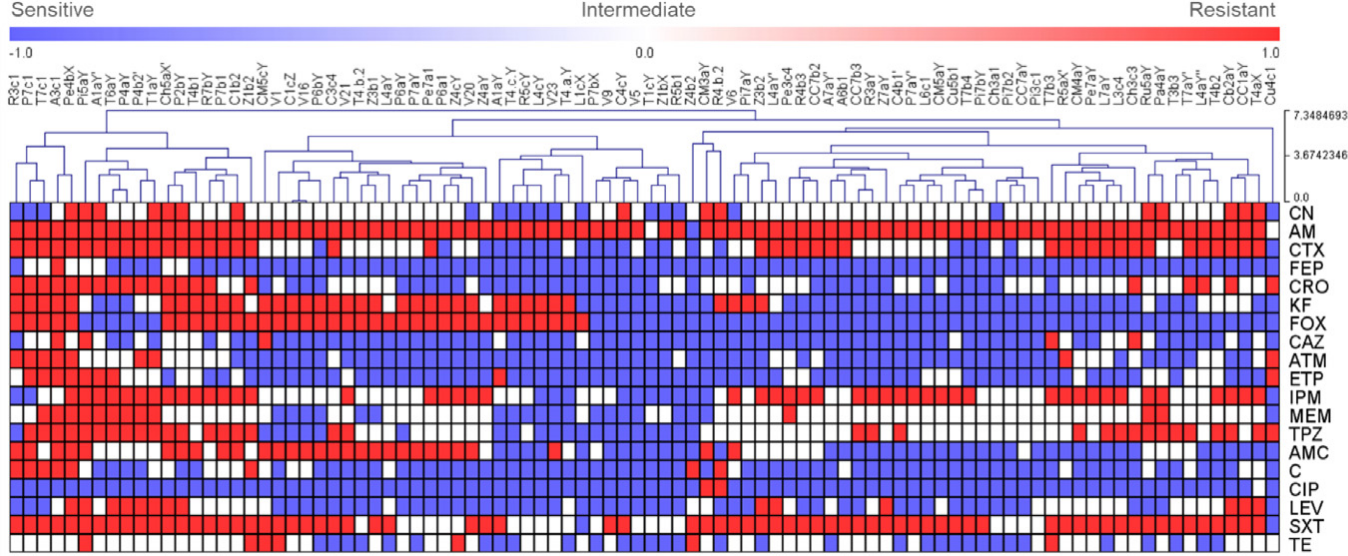
Profiles of antibiotic resistance and a hierarchical tree of other *Enterobacterales* isolates.

**Abbreviations:** Amoxicillin + clavulanic acid 30 mcg, AMC; ampicillin 10 mcg, AM; aztreonam 30 mcg, ATM; cephalothin 30mcg, KF; cefepime 30mcg, FEP; cefotaxime 30mcg, CTX; cefoxitin 30mcg, FOX; ceftazidime 30mcg, CAZ; ceftriaxone 30 mcg, CRO; ciprofloxacin 5mcg, CIP; chloramphenicol 30mcg, C; ertapenem 10 mcg, ETP; gentamicin 10mcg, CN; imipenem 10mcg, IPM; levofloxacin 5mcg, LEV; meropenem 10mcg, MEM; piperacillin/tazobactam 100/10mcg, TPZ; sulfamethoxazole/trimethoprim-b 1.25/23.75, SXT; tetracycline 30mcg, TE.

To visualize the relative similarity of the antimicrobial resistance patterns of the isolates, a hierarchical cluster analysis was performed using the results of the antibiograms, where the phenotypes ‘resistant’, ‘intermediate’, and ‘susceptible’ were observed as red, white, and blue colors respectively. The complete information about the antibiotic resistance profiles and the phenotypic synergies were reported in supplementary Table S3. Figs. 2 and 3 represents the resistance profiles and the hierarchical clustering of *E. coli* and the rest of *Enterobacterales* respectively.

The detection of virulence genes that identify *E. coli* pathotypes (enterotoxigenic ETEC, enteropathogenic EPEC, enteroaggregative EAEC, enteroinvasive EIEC, enterohemorrhagic EHEC) and antibiotic resistance genes that confer resistance to beta-lactams, mobile colistin resistance, carbapenems, quinolones, sulfonamides, and tetracyclines were assessed via PCR. Primers and PCR conditions were listed in supplementary Table S4. Table 1 summarizes the number of potential *E. coli* pathotypes detected and Table 2 the description of the isolates that harbor beta-lactamase resistance genes. The complete information about pathotypes and resistance genes of the 193 isolates were reported in supplementary Table S5.

**Table 1:** Diarrheagenic *E. coli* pathotypes isolated from fresh vegetables.

Sample	Bacteria	Sample ID	Date	Location	*E. coli* Pathotypes (gene)		
					EPEC/EHEC	EHEC	EAEC
					*(eae)*	*(stx2)*	*(pic)*
Lettuce	*E. coli*	L4b1	5/1/2021	Mercado de Productores Agrícolas San Pedro de Riobamba	-	-	+
Tomato	*E. coli*	T5bX	8/3/2021	Mercado La Condamine	-	+	-
White cabbage	*E. coli*	C5b1	8/3/2021	Mercado La Condamine	-	+	-
Radish	*E. coli*	R6c2	5/4/2021	Mercado General Dávalos	+	-	-
Radish	*E. coli*	R3cX	12/4/2021	Mercado La Merced	+	-	-
Total *E. coli* isolates					(2/92)	(2/92)	(1/92)
					2.17%	2.17%	1.09%

**Table 2:** Beta-lactamase resistance genes of *E. coli* isolated from fresh vegetables.

				Location	Beta-lactamases		Carbapenemases	
Sample	Bacteria	Sample ID	Date		*bla*_CTX-M_	*bla*_TEM_	*bla*_SHV_	*bla*_CMY_
Celery	*E. coli*	V11	9/3/2020	Mercado San Francisco	-	-	-	+
Lupine	*E. coli*	V13	9/3/2020	Mercado San Francisco	-	-	-	+
Lettuce	*E. coli*	L4b1	5/1/2021	Mercado de Productores Agrícolas San Pedro de Riobamba	-	-	-	+
Tomato	*E. coli*	T4.c.X	5/1/2021	Mercado de Productores Agrícolas San Pedro de Riobamba	-	+	-	-
Red cabbage	*E. coli*	CM5aX	19/1/2021	Mercado La Condamine	-	-	-	+
Lettuce	*E. coli*	L6bY	1/2/2021	Mercado General Dávalos	-	+	-	-
Red cabbage	*E. coli*	CM3aX	1/2/2021	Mercado La Merced	-	-	-	+
Tomato	*E. coli*	T7aX	8/2/2021	Mercado “El Prado	+	-	-	-
Lettuce	*E. coli*	L4aX	1/3/2021	Mercado “El Prado	-	-	+	-
Total *E. coli* isolates					(1/92)	(2/92)	(1/92)	(5/92)
					1.09%	2.17%	1.09%	5.43%

## 2 Experimental Design, Materials and Methods

### 2.1 Enterobacterales strains

The samples were obtained from markets in the city of Riobamba and processed through an adaptation of the method described previously [1]: 30 grams of fresh and raw vegetables were taken, homogenized with 270 mL of peptone water, and incubated. at 37 °C for 24 hours. Subsequently, streaking was performed on Mac Conkey agar (Merck, Germany), Chromocult (Merck, Germany), and ChromAgar mSuperCARBA (Chromagar, France) which were incubated at 37 °C for 24 hours. One to three colonies with different morphologies of *Enterobacterales* were chosen per sample, to avoid the isolation of repetitive clones. The preliminary identification of the isolated *Enterobacterales* was carried out through biochemical tests: catalase, oxidase, TSI agar, Simmons citrate, lactose test, urea agar, indole production, methyl red test, and Voges-Proskauer. The interpretation of the results was carried out using the Bergey's Manual and the ABIS Automated Biometric Identification System software (https://www.tgw1916.net/bacteria_logare_desktop.html) that executes search tasks by making comparisons in a database that includes several biometric templates.

### 2.2 Phenotypic antibiotic resistance profiles

Antibiograms tested on the strains were determined by the agar disk diffusion method using commercially antibiotic disks (Thermo Scientific™ Oxoid™ and Bioanalyse) on Mueller-Hinton Agar (Thermo Scientific™ Oxoid™) according to the protocols of the U. S. Clinical and Laboratory Institute (CLSI), therefore, the diameter of the zones of inhibition was measured and interpreted as sensible or resistant by referring to CLSI breakpoints [2]

### 2.3 Detection of *E. coli* pathotypes and antibiotic resistance genes detection via PCR

The PCR test was performed according to the standardized protocol of the UTA RAM One Health research group [3,4]: 2.5 µL of DNA from each sample and 22.5 µL of PCR mix containing 12.5 µL DreamTaq PCR Master Mix (ThermoFisher Scientific, USA), 9 µL Nuclease-free water, 0.5 µL Primer 1 and 0.5 µL Primer 2 (final concentration of primers: 0.5 µM) were mixed to run PCR. The PCR conditions were reported in supplementary Table S4. PCR products were analyzed by 1.2% agarose gel electrophoresis stained by Sybr^TM^ Safe DNA Gel Stain (ThermoFisher Scientific, USA).

### 2.4 Heatmaps and hierarchical clustering

Heatmaps and hierarchical clustering were performed using the complete linkage method through Euclidean correlation and sample leaf order optimization [3]. For this purpose, the MeV Multiexperiment Viewer software version 4.8.1 was used [5].

## Ethics Statements

This work does not involve the use of human subjects or animal experiments.

## CRediT authorship contribution statement

**Gabriela Barragán-Fonseca:** Investigation, Formal analysis, Writing – original draft. **Jessica Tubón:** Visualization, Investigation. **William Calero-Cáceres:** Writing – original draft, Formal analysis, Conceptualization, Validation, Writing – review & editing.

## Declaration of Competing Interest

The authors declare that they have no known competing financial interests or personal relationships that could have appeared to influence the work reported in this paper.

## Data Availability

Supplementary Table S5 https://dx.doi.org/10.17632/zffp2cjykv.1 (Original data) (Mendeley Data). Supplementary Table S4 https://dx.doi.org/10.17632/ptgm6jjkfz.1 (Original data) (Mendeley Data). Supplementary Table S3 https://dx.doi.org/10.17632/xs95ww44r9.2 (Original data) (Mendeley Data). Supplementary Table S2 https://dx.doi.org/10.17632/xkvmhxxkt4.1 (Original data) (Mendeley Data). Supplementary Table S1 https://dx.doi.org/10.17632/yh533bg374.1 (Original data) (Mendeley Data).
